# Supplementary material for: Acentrosomal spindles assemble from branching microtubule nucleation near chromosomes in Xenopus laevis egg extract
Source: Nat Commun. 2023 Jun 21;14:3696. doi: 10.1038/s41467-023-39041-z (PMC10284841; doi:10.1038/s41467-023-39041-z)
Supplement: Supplementary file 1 — Supplementary Information [file 41467_2023_39041_MOESM1_ESM.pdf]

# Acentrosomal spindles assemble from branching microtubule nucleation near chromosomes in Xenopus laevis egg extract: supplementary information

Bernardo Gouveia[1†], Sagar U. Setru[2†], Matthew R. King[3], Aaron Hamlin[3], Howard A. Stone[4], Joshua W. Shaevitz[2,5], Sabine Petry[*,3]

[1]Department of Chemical and Biological Engineering
[2]Lewis-Sigler Institute for Integrative Genomics
[3]Department of Molecular Biology
[4]Department of Mechanical and Aerospace Engineering
[5]Department of Physics
Princeton University, Princeton, NJ 08544, USA
†These authors contributed equally.
∗To whom correspondence should be addressed: spetry@princeton.edu

## SUPPLEMENTARY FIGURES

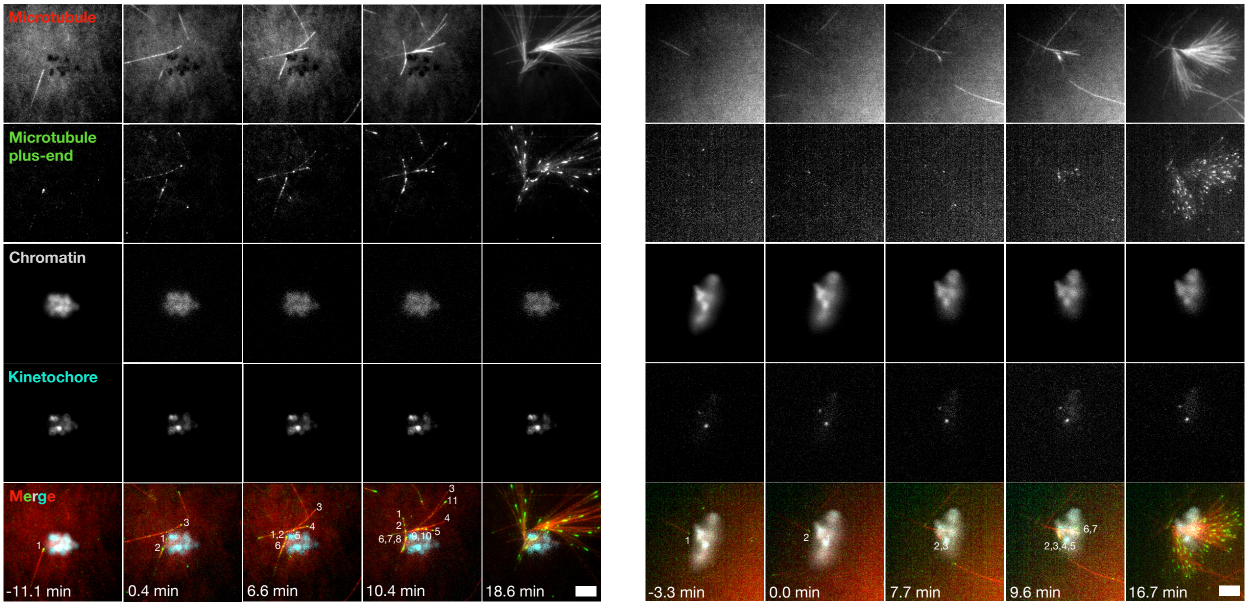


**Figure S1. Additional examples of chromosomal microtubule networks.** $t = 0$ min corresponds to the first nucleation event. Numbers demarcate unique microtubule plus-ends. Scale bars are 5 $\mu$m. Data are representative of 7 extract preparations.

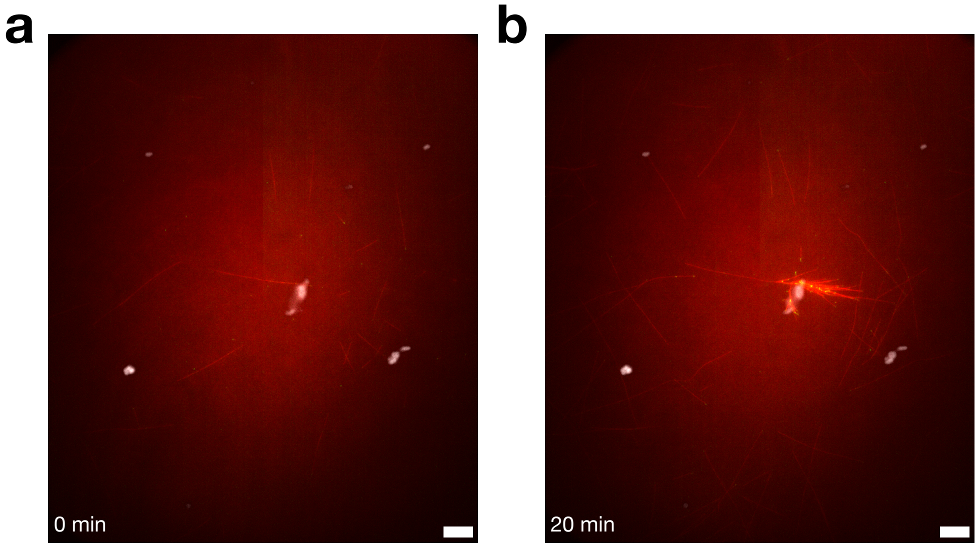


**Figure S2. Starter *de novo* microtubules randomly nucleate to initiate microtubule-dependent microtubule networks near chromosomes.** Snapshots shown at (a) onset of first microtubule-dependent nucleation event and (b) 20 minutes later. Scale bars are 10 $\mu$m. Data are representative of 7 extract preparations.

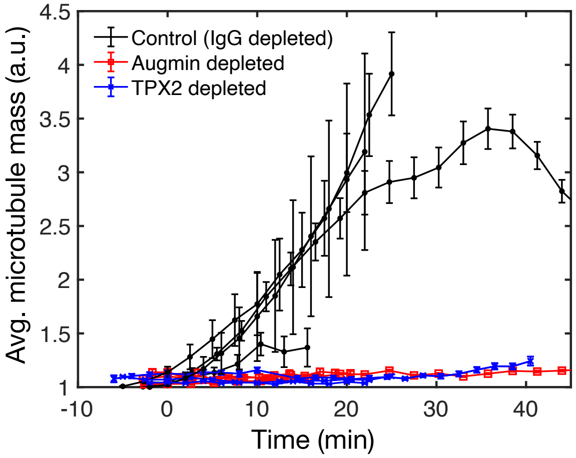


**Figure S3. Average microtubule mass over time for chromosomal microtubule networks in control depleted (black), augmin depleted (red), and TPX2 depleted (blue) extracts.** Each curve represents an independent extract experiment. Error bars are generated by averaging over multiple chromosome clusters and are plotted as standard error of the mean. Chromosome clusters that generated networks in the control conditions were included and all chromosome clusters in the depletion conditions were included. Only one chromosome cluster showed microtubule network formation in either depletion condition (< 1%, see Fig. 2b). $n = 19.7 \pm 19.8$ chromosome clusters were considered per curve (mean $\pm$ standard deviation).

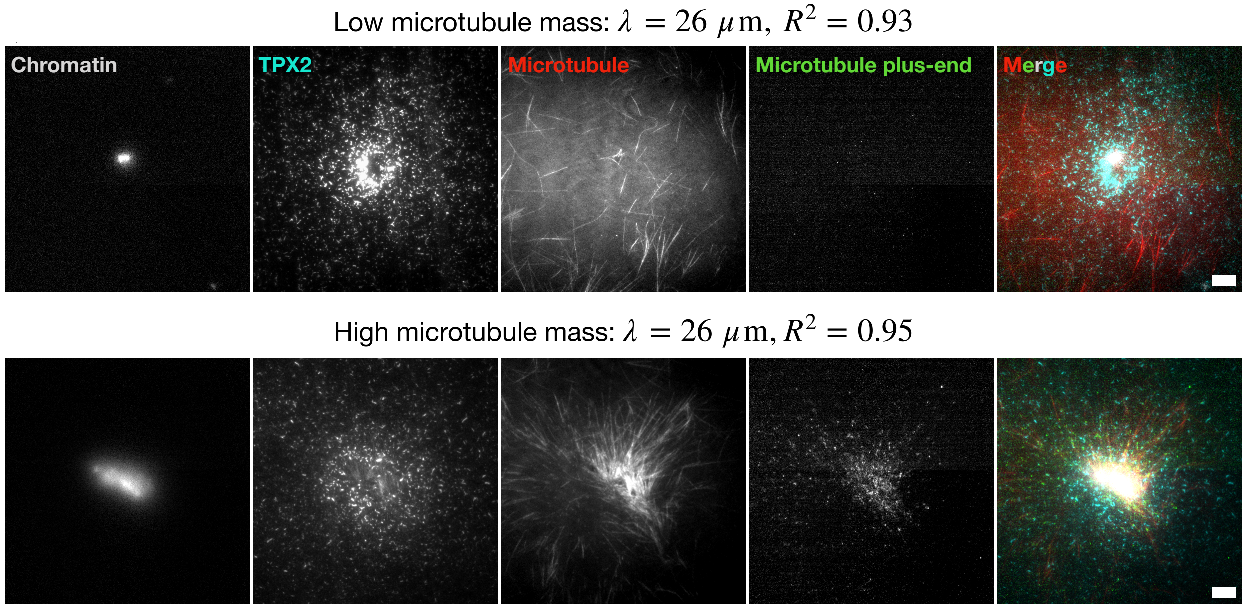


**Figure S4. Measurement of SAF gradient length scale is insensitive to the number of microtubules nucleated.** The top images show a chromosome that nucleated relatively few microtubules in its vicinity, whereas the bottom images show a chromosome that nucleated a dense microtubule network. Even though there are qualitative differences, the estimation of $\lambda$ is unaffected, and therefore the dominant feature measured from these experiments is the length scale of the free, unbound GFP-TPX2 gradient. Scale bars are 5 $\mu$m. Data are from 7 chromosome clusters across 2 extract preparations**.**

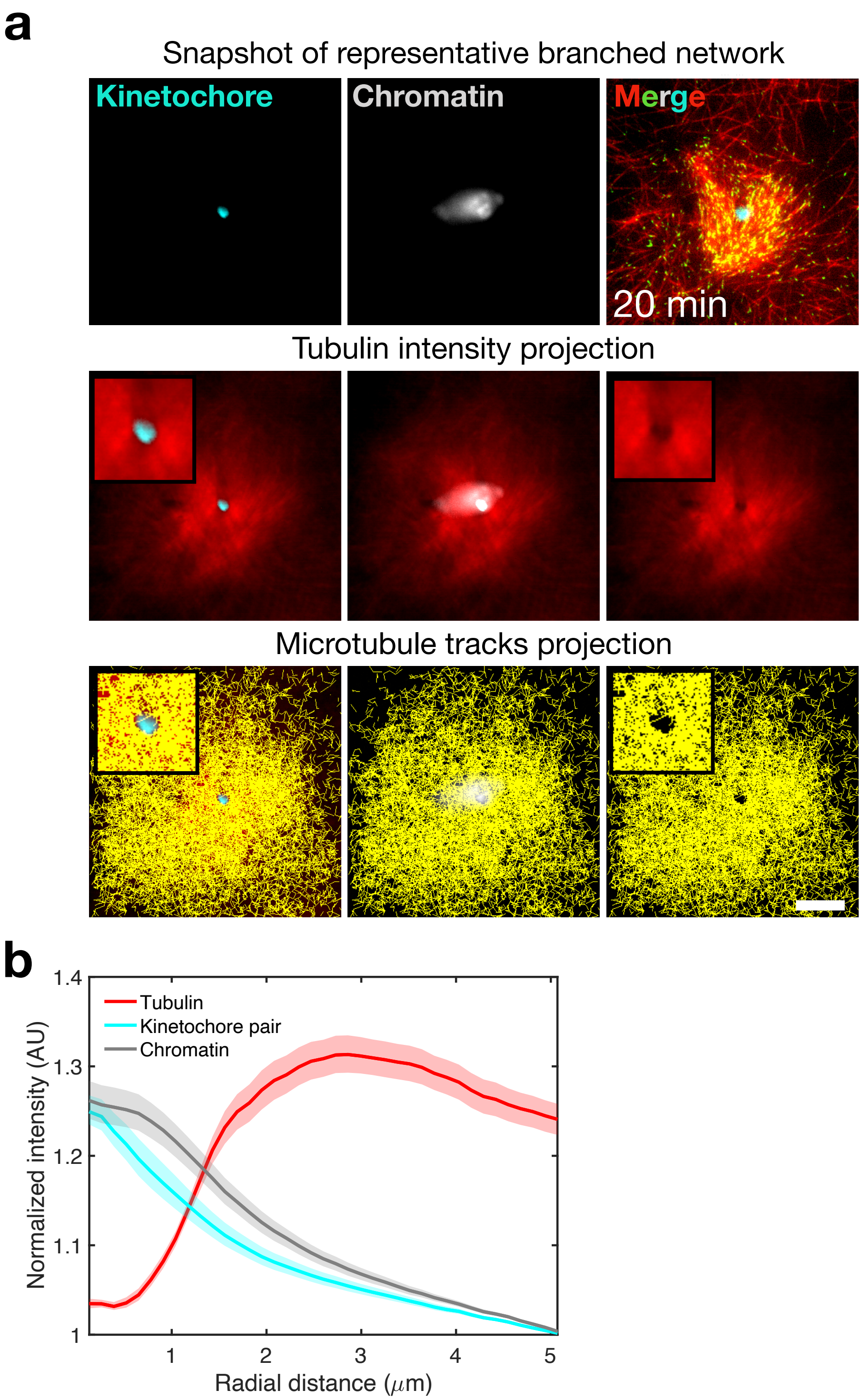


**Figure S5. Kinetochores act as a void for microtubules.** (a) Images showing a snapshot of the representative chromosomal branched network under analysis (top row), tubulin intensity projection averaged over time (middle row), and microtubule tracks projection averaged over time

in the vicinity of the chromosome (bottom row). Both the tubulin and microtubule tracks projections exhibit a void at the kinetochore because microtubules cannot polymerize through kinetochores in our system. Scale bars are 10 $\mu$m. Zoomed-in insets of the void region are 10 $\mu$m x 10 $\mu$m. (b) Quantification of mean normalized intensity for kinetochore, tubulin, and chromatin channels. Data are from the 3 chromosome clusters across 2 extracts that had only 1 void visible. Every chromosome cluster featured at least 1 void. Shaded error bands represent 95% bootstrap confidence intervals.

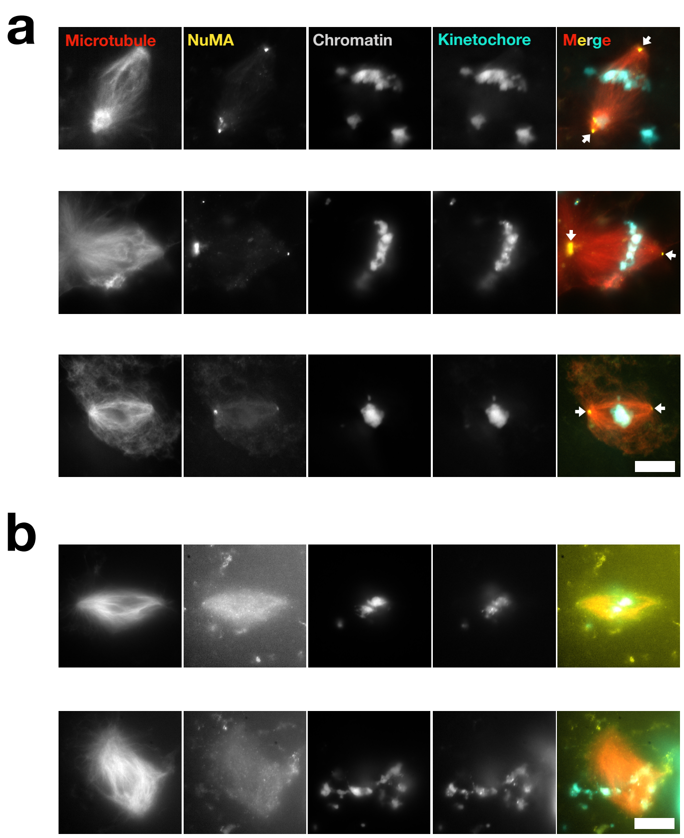


**Figure S6. Additional examples of acentrosomal bipolar spindles in bulk extract.** (a) White arrows mark the NuMA labeled poles. Scale bars are 10 $\mu$m. (b) Negative control immunostaining of bipolar spindles with a random IgG showing no distinct or significant spindle localization. Scale bars are 10 $\mu$m. Data are from $n = 173$ spindles across 13 different extract preparations.

## SUPPLEMENTARY MOVIE LEGENDS

**Movie S1. Branching microtubule nucleation at kinetochores with vanadate.** The field is 30 $\mu$m x 30 $\mu$m.

**Movie S2. Branched microtubule network formation in control depleted (left) and augmin depleted (right) extracts**. The fields are 140 $\mu$m x 166 $\mu$m.

**Movie S3. Branched microtubule network formation in control depleted (left) and TPX2 depleted (right) extracts**. The fields are 140 $\mu$m x 166 $\mu$m.

**Movie S4. Branching microtubule nucleation in a uniform field of SAFs.** The field is 34 $\mu$m x 22 $\mu$m.

**Movie S5. Branching microtubule nucleation at kinetochores with motor activity.** The field is 30 $\mu$m x 30 $\mu$m.

**Movie S6. Bipolar spindle assembly around chromosomes.** The field is 40 $\mu$m x 40 $\mu$m.

# 1 Theory - Derivation

## 1.1 Dynamic instability

The first step of our theory is to introduce a model for the dynamic instability of microtubules. Following the now standard approach [1], we define $m\,(t,x,\ell)$ ($m_{\rm s}\,(t,x,\ell)$) as the probability density of observing a growing (shrinking) microtubule of length $\ell$ with minus end at $x$ at time $t$. We adopt the physiological picture that microtubules grow (shrink) from their plus end at speed $U$ ($U_{\rm s}$) and are nucleated and stabilized at their minus end. A growing (shrinking) microtubule can stochastically transition into a shrinking (growing) microtubule at a frequency $f_{\rm c}$ ($f_{\rm r}$) called the catastrophe (rescue) frequency. This entire process occurs far from equilibrium and is regulated by the rate at which GTP-tubulin is hydrolyzed into GDP-tubulin [2]. Because $\langle\ell\rangle/U \ll \langle\ell\rangle^2/D_{\rm M}$ is easily satisfied [3], where $\langle\ell\rangle$ is the average microtubule length and $D_{\rm M}$ is the thermal diffusivity of a microtubule, we may neglect diffusive transport of microtubules. We do not consider active transport by motors in this theory. Therefore, the master equations for the probability densities in the continuum limit are

$$\partial_t m + U\partial_\ell m = f_{\rm r} m_{\rm s} - f_{\rm c} m \tag{1a}$$
$$\partial_t m_{\rm s} - U_{\rm s}\partial_\ell m_{\rm s} = -f_{\rm r} m_{\rm s} + f_{\rm c} m, \tag{1b}$$

which are of course only valid on a length scale greater than multiple tubulin dimers.

We now make two simplifying assumptions. Firstly, we utilize the fact that rescue events are rare and take $f_{\rm r} = 0$ [4, 5]. That is, once a growing microtubule catastrophes, it will inevitably shrink to zero length. Secondly, we take advantage of the fact that $U_{\rm s} \gg U$ [6]. That is, microtubules shrink much faster than they grow, and so we take them to effectively disappear once they catastrophe. Under these conditions equations (1) reduce to

$$\partial_t m + U\partial_\ell m = -f_{\rm c} m. \tag{2}$$

The essential feature of equation (2) is the eventual enforcement of a bounded microtubule length distribution: $m(t \to \infty, x, l) \sim \exp\left(-\ell/\langle\ell\rangle\right)$, where $\langle\ell\rangle = U/f_{\rm c}$ is the average microtubule length. Such exponential distributions have indeed been experimentally verified [3, 7, 8]. Closure of equation (2) requires an initial condition, which we take to be $m\,(t=0,x,\ell) = 0$, as well as a specification of how new microtubules are nucleated in time and space, which we shall encode in $m\,(t,x,\ell=0)$.

## 1.2 Microtubule nucleation

We now introduce our model for branching microtubule nucleation regulated by the RanGTP pathway. Branching nucleation, whereby new microtubules nucleate off of preexisting ones, is catalyzed by proteins effectors that bind to microtubules, some of which

(e.g. TPX2 [9–11]) are spatially regulated by the RanGTP pathway and are referred to as a spindle assembly factors (SAFs) [12]. At the onset of spindle assembly, SAFs are sequestered by so-called importin proteins [13]. They are freed to participate in nucleation processes only when RanGTP binds to the importin-SAF complex, which releases the SAF. RanGTP is produced at chromosomes through the RCC1 pathway and released into the cytoplasm, where it can either bind to importin-SAF complexes or be hydrolyzed into its inactive RanGDP form. Thus, RanGTP exists as a gradient around chromosomes.

The simplest model of branching nucleation one can posit is that a branched microtubule is most likely to nucleate wherever the concentration of SAFs bound to preexisting microtubules is highest. Hence, the probability of nucleating a branched microtubule is proportional to the local concentration of bound SAFs, which we denote by $c_{\mathrm{b}}(t,x)$. We note that in reality multiple factors must bind to nucleate a branch [8, 11, 14], some of which are not spatially regulated by RanGTP. However, SAFs bind first [8]. Therefore our model is only strictly valid if the binding of SAFs is rate-limiting. However, taking into account multiple binding events does not affect the main qualitative conclusions of this work [8]. Given these considerations we write the nucleation condition as

$$m(t,x,\ell=0) = \frac{1}{\langle\ell\rangle}\left(\delta(x) + c_{\mathrm{b}}(t,x)\right), \tag{3}$$

where $\delta(x)$ is the Dirac delta function. The first term captures the initial nucleation of a *de novo* microtubule at $x=0$ and the second term represents the contribution due to branching nucleation. The prefactor $1/\langle\ell\rangle$ sets the units so that $M(t,x) = \int_0^\infty \mathrm{d}\ell\ m(t,x,\ell)$ measures the number density of minus ends and $P(t,x) = \int_0^\infty \mathrm{d}\ell\ m(t,x-\ell,\ell)$ measures the number density of plus ends.

To close the problem, we must express $c_{\mathrm{b}}$ in terms of $m$ through the reaction-diffusion network regulated by RanGTP (Fig. 4a). It is not our goal here to model the delicate intricacies of the full chemical kinetics involved in the RanGTP pathway [13, 15]. Rather, we seek the simplest tractable description that will preserve the essential microtubule behavior. In this spirit, we first assume the importin binding kinetics are fast enough to come to local equilibrium, which allows us to write the algebraic relation

$$k_{\mathrm{I}}c\,[\mathrm{Importin-SAF}] = k_{\mathrm{I}}^{(\mathrm{r})}\,[\mathrm{Importin-RanGTP}]\,c_{\mathrm{u}}, \tag{4}$$

where $c$ is the concentration of free RanGTP and $c_{\mathrm{u}}$ is the concentration of unbound SAFs. Therefore we have that $c_{\mathrm{u}} = \left(k_{\mathrm{I}}/k_{\mathrm{I}}^{(\mathrm{r})}\right)\left([\mathrm{Importin-SAF}]/[\mathrm{Importin-RanGTP}]\right)c := K_{\mathrm{I}}c$, where we define $K_{\mathrm{I}}$ to be a constant that encodes all the kinetic activity of the importin molecules. Hence, in this approximation the unbound SAF concentration $c_{\mathrm{u}}$ is simply proportional to the RanGTP concentration $c$. Together with equation (4), we find that $c$ satisfies the reaction-diffusion equation

$$\partial_t c = D\partial_x^2 c - k_{\mathrm{H}}c, \tag{5}$$

where $D$ is the diffusivity of RanGTP and $k_{\mathrm{H}}$ is the hydrolysis rate of RanGTP $\to$ RanGDP. We now assume that protein diffusion is fast compared to microtubule dynamics, i.e. $\langle l\rangle^2/D \ll \langle l\rangle/U$, so that the RanGTP gradient is quasistatic with respect to the microtubule dynamics [4, 5]. In this limit equation (5) becomes $0 = \frac{\mathrm{d}^2 c}{\mathrm{d}x^2} - k_{\mathrm{H}} c$. Enforcing the boundary conditions $-D\frac{\mathrm{d}c}{\mathrm{d}x}(x = d) = J$ and $c(|x| \to \infty) \to 0$, where $J$ is the flux of RanGTP into the cytoplasm produced by chromosomes, furnishes the solution $c(x) = c_0 \exp(-|x-d|/\lambda)$, where $c_0 = J\lambda/D$ is the maximum RanGTP concentration at $x = 0$, $\lambda = \sqrt{D/k_{\mathrm{H}}}$ is the characteristic length scale of the RanGTP/SAF gradient, and $d$ is the distance between the chromosome and the minus end of the initial *de novo* mother microtubule. Hence the concentration profile of unbound SAFs satisfies

$$c_{\mathrm{u}}(x) = K_{\mathrm{I}} c_0 e^{-|x-d|/\lambda}. \tag{6}$$

We now obtain the concentration of bound SAFs $c_{\mathrm{b}}$ needed to close the nucleation condition (3). The simplest way to proceed is to assume that the binding of SAFs to a microtubule occurs at local equilibrium, so that $\kappa_{\mathrm{on}} c_{\mathrm{u}} \mathcal{F}[m, x] = \kappa_{\mathrm{off}} c_{\mathrm{b}}$, or

$$c_{\mathrm{b}}(t, x) = K c_{\mathrm{u}}(x) \mathcal{F}[m, x] \tag{7}$$

where $K = \kappa_{\mathrm{on}}/\kappa_{\mathrm{off}}$ is the ratio between on and off rates of SAFs binding to and unbinding from a microtubule. $\mathcal{F}[m, x]$ is a functional that expresses the concentration of microtubule lattice at $x$ that can serve as a binding site for unbound SAFs at $x$, given the minus end distribution $m(t, x', \ell)$. We can write this functional as

$$\mathcal{F}[m, x] = \int_0^\infty \mathrm{d}\ell \int_0^x \mathrm{d}x' \; \Theta(\ell - (x - x'))\, m(t, x', \ell) \tag{8}$$

where $\Theta$ is the Heaviside step function. The structure of equation (8) ensures that a microtubule with minus end at $x'$ can nucleate a branched microtubule at $x$ so long as its length $\ell$ is such that $\ell > x - x'$. This introduces strong nonlocality to the mathematical formalism and allows us to describe the evolution and variation of the resulting branched network on length scales comparable to and even smaller than the average microtubule length. This generalizes previous continuum theories which were only valid over length scales larger than the average microtubule length [3, 16, 17]. At this stage we have formulated the theory in one spatial dimension, which is justified by the fact that branching angles are shallow, which ensures that the resulting branched networks are highly polar.

## 1.3 Rescaling and problem statement

We now rescale equations (2), (3), and (7) using the dimensionless variables $T = tf_{\mathrm{c}}$, $X = x/\langle l\rangle$, $L = l/\langle l\rangle$, and $\psi = m\langle l\rangle^2$ to arrive at the dimensionless problem statement

$$\partial_T \psi + \partial_L \psi = -\psi \tag{9a}$$

$$\psi(T = 0, X, L) = 0 \tag{9b}$$

$$\psi(T, X, L = 0) = \delta(X) + \mathcal{B} e^{-\left|\frac{X-D}{\Lambda}\right|} \mathcal{F}[\psi, X], \tag{9c}$$

where $\mathcal{F}[\psi, X] = \int_0^\infty \mathrm{d}L' \int_0^X \mathrm{d}X' \, \Theta(L' - (X - X'))\, \psi(T, X', L')$. We identify the number $\mathcal{B} = KK_I c_0 \langle l \rangle = KK_I c_0 U / f_\mathrm{c}$ as representing the competition between how many new microtubule branches nucleate versus how many microtubules are lost to catastrophes. Hence we refer to $\mathcal{B}$ as the *branching number*. Note that in $n$ spatial dimensions we would have $\mathcal{B} = KK_I c_0 \langle l \rangle^n$. Additionally, two geometric ratios appear. $\Lambda = \lambda / \langle l \rangle$ is the ratio of the SAF gradient length scale to the average microtubule length, and $D = d/\langle l \rangle$ is the ratio of the distance between the initial *de novo* nucleation event and the chromosome to the average microtubule length.

Solution of this partial integrodifferential equation for the distribution $\psi$ will establish how dynamic microtubules are organized by branching nucleation processes spatially regulated by chromosomes, and hence offer insight into early spindle assembly.

# 2 Theory - Branching in a uniform field of SAFs

We first consider the case where the concentration of SAFs is uniform, which corresponds to the limit $\Lambda \to \infty$ in equations (9). There are two reasons to work in this limit. Firstly, we can perform experiments using a non-hydrolyzable mutant version of RanGTP, RanQ69L, that produces a uniform field of SAFs in *X. laevis* extract, allowing us to benchmark our model on a simpler system (Fig. 3). Secondly, the model has an exact closed-form solution in this limit, which will give us analytical insight into the organization of these dynamic branched networks.

In this limit, the spatial bias imposed by the SAF gradient vanishes. Therefore the kernel of $\mathcal{F}$ has translational invariance, which motivates solution by Laplace transform. We define $\tilde{\psi} = \int_0^\infty \mathrm{d}T \; e^{sT} \psi(T, X, L)$. Taking the Laplace transform of equation (9a), using the initial condition (9b), and integrating in $L$ gives

$$\tilde{\psi} = \tilde{\psi}(X, L = 0; s)\, e^{-(s+1)L}. \tag{10}$$

We get $\tilde{\psi}(X, L = 0; s)$ by Laplace transforming the nucleation condition (9c), resulting in the double integral equation

$$e^{(s+1)L}\tilde{\psi} - \mathcal{B}\mathcal{F}\left[\tilde{\psi}, X\right] = \frac{\delta(X)}{s}. \tag{11}$$

To make progress, we define the spatial Laplace transform of $\tilde{\psi}$ as $\hat{\tilde{\psi}} = \int_0^\infty \mathrm{d}X \; e^{-kX} \tilde{\psi}(X, L; s)$. Taking the spatial Laplace transform of equation (11) gives

$$e^{(s+1)L}\hat{\tilde{\psi}} - \mathcal{B} \int_0^\infty \mathrm{d}L' \int_0^\infty \mathrm{d}X \int_0^X \mathrm{d}X' \; e^{-kX} \Theta(L' - (X - X'))\, \tilde{\psi}(X', L'; s) = \frac{\delta(X)}{s} \tag{12}$$

Swapping integration orders on $X$ and $X'$ factors the spatial integrals into a product of two spatial Laplace transforms in $X'$ and $X - X'$, the latter of which may be explicitly

computed. The result is the single integral equation

$$e^{(s+1)L}\hat{\tilde{\psi}} - \frac{\mathcal{B}}{k}\int_0^\infty \mathrm{d}L' \left(1 - e^{-kL'}\right)\hat{\tilde{\psi}}\left(L'; s, k\right) = \frac{1}{s}, \tag{13}$$

for which the solution may be obtained by proposing the anzats $e^{(s+1)L}\hat{\tilde{\psi}} = \frac{\mathcal{B}}{k}c(s,k) + \frac{1}{s}$ for a to be determined function $c(s,k)$ [18]. Substituting this anzats into equation (13) and preforming the necessary integrations gives $c = \frac{k}{s(s+1)(s+k+1)-s\mathcal{B}}$, and so we find

$$\hat{\tilde{\psi}} = \left\{\frac{\mathcal{B}}{(s+1)(s+k+1)} + 1\right\}\frac{1}{s}e^{-(s+1)L}. \tag{14}$$

The inverse spatial Laplace transform of equation (14) may be computed and expressed in terms of elementary functions, giving the result

$$\tilde{\psi} = \left\{\frac{\mathcal{B}}{s+1}e^{\left[\frac{\mathcal{B}}{s+1}-(s+1)\right]X} + \delta\left(X\right)\right\}\frac{1}{s}e^{-(s+1)L}. \tag{15}$$

From equation (15) we may directly compute the dimensionless plus end distribution $\Phi = \int_0^\infty \mathrm{d}L\ \psi(T, X-L, L) \implies \tilde{\Phi} = \int_0^\infty \mathrm{d}L\ \tilde{\psi}(X-L, L; s)$, which is the main quantity of interest to us since we can directly track microtubule plus ends. Performing the integration and neglecting the redundant $\mathcal{B} = 0$ solution gives

$$\tilde{\Phi} = \frac{1}{s}e^{\left[\frac{\mathcal{B}}{s+1}-(s+1)\right]X} \tag{16}$$

for the plus end distribution. $\tilde{\Phi}$ has a simple pole at $s = 0$ and an essential singularity at $s = -1$. Such essential singularities often manifest whenever there are strongly nonlocal physics, such as in the Kosterlitz-Thouless transition [19]. The essential singularity renders the principal part of the local Laurent expansion infinite, precluding the application of standard residue calculus [20]. Therefore, the inverse Laplace transform of equation (16) cannot be computed exactly in terms of known functions, so we employ the Bromwich integral representation

$$\Phi = \frac{1}{2\pi i}\int_{-i\infty}^{i\infty} \mathrm{d}s\ \frac{1}{s}e^{sT+\left[\frac{\mathcal{B}}{s+1}-(s+1)\right]X}, \tag{17}$$

where the notation means we integrate along a vertical line in the complex $s$ plane. Note that we have let the integration path tend towards the imaginary axis, since $s = 0$ is just a simple pole, and so the integral is to be interpreted in the principal value sense. Thus, we have derived an exact formula for the plus end distribution of dynamic microtubules branching in a uniform field of SAFs.

We first ask whether the plus end distribution $\Phi$ can attain a bounded distribution, which would imply the existence of statistically stationary branched networks. Recall

that the final value theorem states $\lim_{T\to\infty}\Phi(T,X) = \lim_{s\to 0} s\tilde{\Phi}(X,s)$ so long as $|\Phi|$ is bounded as $T \to \infty$. Application of the final value theorem to equation (16) immediately gives

$$\Phi \to e^{(\mathcal{B}-1)X} \text{ for } T \to \infty,\ \mathcal{B} < 1, \tag{18}$$

where we require $\mathcal{B} < 1$ since $e^{(\mathcal{B}-1)X}$ is no longer bounded for all $X$ if $\mathcal{B} > 1$. The case where $\mathcal{B} = 0$ recovers the known result that dynamic microtubules nucleating from a local source have exponentially distributed lengths in the bounded growth regime [1]. The physical interpretation of the distribution (18) is simple: when $\mathcal{B} < 1$, more microtubules are lost to catastrophes than are produced via branching nucleation. Hence, the branched network can only propagate a finite distance before reaching a steady state (Fig. 3d).

The case of $\mathcal{B} = 1$ is subtle. The final value theorem (18) would suggest that $\Phi \to 1$, but this is not the whole story. Upon numerically evaluating the inverse Laplace transform (17) for $\mathcal{B} = 1$ using de Hoog's method [21, 22], we see that the solution represents traveling waves of constant microtubule density, and therefore no steady state exists (Fig. 3e). In this regime, microtubules are made via branching nucleation as often as they are lost to catastrophes, and so the system can propagate indefinitely while sustaining a constant density. We computed the position of the inflection point of $\Phi(T,X)$ versus time, $X^*(T)$, and found a linear relationship, confirming that these profiles indeed correspond to traveling waves. Interestingly, the system selects a wave speed of $V \simeq U/2$. Traveling waves of constant microtubule density have been observed in the study of branching asters [16], where the authors provide a similar physical interpretation of the waves, as well as simulations of the traveling microtubule density profiles. Here, we see that their results are a special case of our theory when $\mathcal{B} = 1$, and furthermore, we provide an exact, parameter free formula for such profiles via equation (17).

When $\mathcal{B} > 1$, the production of new microtubule branches out competes the loss of microtubules due to catastrophes, and so a branched network forms that propagates outwards and grows autocatalytically with time (Fig. 3f). Interestingly, the position of the maximum of this network, $X_{\text{max}}(T)$, also increases linearly with time at a wave speed of $V \simeq U/2$. We see that our experimental branched networks fall in this regime for the first $\sim 10$ min with $\mathcal{B} = 1.7$ (Fig. 3c). However, after this autocatalytic growth, the experimental branched network saturates in a way consistent with a limiting number of available nucleators.

# 3 Theory - Branching in a SAF gradient

We now consider the full problem of microtubules branching in SAF gradient described by equations (9). Because the SAF gradient profile $C_{\text{u}}(X) = \exp\left(-|\frac{X-D}{\Lambda}|\right)$ breaks the translational invariance of the problem, an exact closed-form solution is no longer possible. Furthermore, regular perturbation methods are ill-suited for systems that exhibit autocatalytic growth. Hence, we proceed to solve the complete equations (9) numerically.

We begin by working with the Laplace transformed distribution $\tilde{\psi}$. In the case where there is a SAF gradient, equation (11) becomes

$$e^{(s+1)L}\tilde{\psi} - \mathcal{B}C_{\mathrm{u}}(X)\mathcal{F}\left[\tilde{\psi}, X\right] = \frac{\delta\left(X\right)}{s}. \tag{19}$$

We now substitute the ansatz

$$\tilde{\psi} = \left[\frac{\delta(X)}{s} + \tilde{y}\right] e^{-(s+1)L} \tag{20}$$

into equation (19) for a to be determined function $\tilde{y} = \tilde{y}(X;s)$. Evaluating the integrals appear upon this substitution results in

$$\tilde{y} - \mathcal{B}C_{\mathrm{u}}(X)\int_0^\infty \mathrm{d}L\ e^{-(s+1)L}\int_0^X \mathrm{d}X'\ \Theta(L-(X-X'))\tilde{y}(X') = \frac{\mathcal{B}C_{\mathrm{u}}(X)e^{-(s+1)X}}{s(s+1)}, \tag{21}$$

which is an integral equation for $\tilde{y}(X;s)$. In order to solve equation (21) numerically, we discretize all variables onto a regular grid $X \to X_i$ and $\tilde{y}(X;s) \to \tilde{y}_i(s)$ for $i = 0, ..., M$ and we choose $\Delta X = X_{i+1} - X_i = 0.1$. We first evaluate the integral over $X'$ using the trapezoidal rule and then evaluate the integral over $L$ analytically. The result is

$$\tilde{y}_i - \frac{\mathcal{B}C_{\mathrm{u}}(X_i)\Delta X}{s+1}\left[\frac{e^{-(s+1)X_i}}{2}\tilde{y}_0 + \sum_{m=1}^{i-1} e^{-(s+1)(X_i-X_m)}\tilde{y}_m + \frac{1}{2}\tilde{y}_i\right] = \frac{\mathcal{B}C_{\mathrm{u}}(X_i)e^{-(s+1)X_i}}{s(s+1)}. \tag{22}$$

Equation (22) has the structure of the linear system $(\delta_{im} - w_{im})\tilde{y}_m = b_i$ (Einstein summation convention is used here), which we invert using standard LU decomposition to find the desired $\tilde{y}_i(s)$.

With $\tilde{y}_i(s)$ now constructed, we numerically compute the inverse Laplace transform using de Hoog's method [21, 22] to generate $y_i(T)$, which allows us to compute $\psi(T, X_i, L)$ using equation (20). The desired plus end distribution is computed from $\Phi(T, X_i) = \int_0^\infty \mathrm{d}L\ \psi(T, X_i - L, L)$ using linear interpolation and the trapezoidal rule. We benchmarked our numerical method using $C_{\mathrm{u}}(X) = 1$ to ensure that it reproduced the results of the uniform field branched networks.

We find that the parameter choice of $\mathcal{B} = 2$ best reproduces the plus-end distribution of the experimental branched networks that form around chromosomes (Fig. 4c), using the experimentally measured value of $\Lambda = 3$ (Fig. 4b).

# References


[1] Marileen Dogterom and Stanislas Leibler. Physical aspects of the growth and regulation of microtubule structures. *Physical review letters*, 70(9):1347, 1993.

[2] Thomas CT Michaels, Shuo Feng, Haiyi Liang, and L Mahadevan. Mechanics and kinetics of dynamic instability. *Elife*, 9:e54077, 2020.

[3] Franziska Decker, David Oriola, Benjamin Dalton, and Jan Brugués. Autocatalytic microtubule nucleation determines the size and mass of xenopus laevis egg extract spindles. *Elife*, 7:e31149, 2018.

[4] R Wollman, EN Cytrynbaum, JT Jones, T Meyer, JM Scholey, and A Mogilner. Efficient chromosome capture requires a bias in the 'search-and-capture'process during mitotic-spindle assembly. *Current Biology*, 15(9):828–832, 2005.

[5] Raja Paul, Roy Wollman, William T Silkworth, Isaac K Nardi, Daniela Cimini, and Alex Mogilner. Computer simulations predict that chromosome movements and rotations accelerate mitotic spindle assembly without compromising accuracy. *Proceedings of the National Academy of Sciences*, 106(37):15708–15713, 2009.

[6] Christopher Gell, Volker Bormuth, Gary J Brouhard, Daniel N Cohen, Stefan Diez, Claire T Friel, Jonne Helenius, Bert Nitzsche, Heike Petzold, Jan Ribbe, et al. Microtubule dynamics reconstituted in vitro and imaged by single-molecule fluorescence microscopy. In *Methods in cell biology*, volume 95, pages 221–245. Elsevier, 2010.

[7] Jan Brugués, Valeria Nuzzo, Eric Mazur, and Daniel J Needleman. Nucleation and transport organize microtubules in metaphase spindles. *Cell*, 149(3):554–564, 2012.

[8] Akanksha Thawani, Howard A Stone, Joshua W Shaevitz, and Sabine Petry. Spatiotemporal organization of branched microtubule networks. *eLife*, 8:e43890, 2019.

[9] Raymundo Alfaro-Aco, Akanksha Thawani, and Sabine Petry. Biochemical reconstitution of branching microtubule nucleation. *eLife*, 9, 2020.

[10] Matthew R King and Sabine Petry. Phase separation of tpx2 enhances and spatially coordinates microtubule nucleation. *Nature Communications*, 11(1):1–13, 2020.

[11] Sagar U Setru, Bernardo Gouveia, Raymundo Alfaro-Aco, Joshua W Shaevitz, Howard A Stone, and Sabine Petry. A hydrodynamic instability drives protein droplet formation on microtubules to nucleate branches. *Nature Physics*, 17(4):493–498, 2021.

[12] Petr Kalab and Rebecca Heald. The rangtp gradient–a gps for the mitotic spindle. *Journal of cell science*, 121(10):1577–1586, 2008.

[13] Maïwen Caudron, Gertrude Bunt, Philippe Bastiaens, and Eric Karsenti. Spatial coordination of spindle assembly by chromosome-mediated signaling gradients. *Science*, 309(5739):1373–1376, 2005.

[14] Sabine Petry, Aaron C Groen, Keisuke Ishihara, Timothy J Mitchison, and Ronald D Vale. Branching microtubule nucleation in xenopus egg extracts mediated by augmin and tpx2. *Cell*, 152(4):768–777, 2013.

[15] Chaitanya A Athale, Ana Dinarina, Maria Mora-Coral, Céline Pugieux, Francois Nedelec, and Eric Karsenti. Regulation of microtubule dynamics by reaction cascades around chromosomes. *Science*, 322(5905):1243–1247, 2008.

[16] Keisuke Ishihara, Kirill S Korolev, and Timothy J Mitchison. Physical basis of large microtubule aster growth. *Elife*, 5:e19145, 2016.

[17] Bryan Kaye, Olivia Stiehl, Peter J Foster, Michael J Shelley, Daniel J Needleman, and Sebastian Fürthauer. Measuring and modeling polymer concentration profiles near spindle boundaries argues that spindle microtubules regulate their own nucleation. *New Journal of Physics*, 20(5):055012, 2018.

[18] Subrahmanyan Chandrasekhar. *Radiative transfer*. Courier Corporation, 2013.

[19] John Michael Kosterlitz and David James Thouless. Ordering, metastability and phase transitions in two-dimensional systems. *Journal of Physics C: Solid State Physics*, 6(7):1181, 1973.

[20] George B Arfken and Hans J Weber. *Mathematical methods for physicists*. American Association of Physics Teachers, 1999.

[21] Frank R De Hoog, JH Knight, and AN Stokes. An improved method for numerical inversion of laplace transforms. *SIAM Journal on Scientific and Statistical Computing*, 3(3):357–366, 1982.

[22] Fredrik Johansson et al. *mpmath: a Python library for arbitrary-precision floating-point arithmetic (version 0.18)*, December 2013. `http://mpmath.org/`.
