# Supplementary material for: Acentrosomal spindles assemble from branching microtubule nucleation near chromosomes in Xenopus laevis egg extract
Source: Nat Commun. 2023 Jun 21;14:3696. doi: 10.1038/s41467-023-39041-z (PMC10284841; doi:10.1038/s41467-023-39041-z)
Supplement: Supplementary file 3 — Reporting Summary [file 41467_2023_39041_MOESM3_ESM.pdf]

nature portfolio

Corresponding author(s): Sabine Petry

Last updated by author(s): Apr 24, 2023

Reporting Summary

Nature Portfolio wishes to improve the reproducibility of the work that we publish. This form provides structure for consistency and transparency in reporting. For further information on Nature Portfolio policies, see our Editorial Policies and the Editorial Policy Checklist.

Statistics

For all statistical analyses, confirm that the following items are present in the figure legend, table legend, main text, or Methods section.

n/a	Confirmed	
☐	☒	The exact sample size (n) for each experimental group/condition, given as a discrete number and unit of measurement
☐	☒	A statement on whether measurements were taken from distinct samples or whether the same sample was measured repeatedly
☐	☒	The statistical test(s) used AND whether they are one- or two-sided<br>*Only common tests should be described solely by name; describe more complex techniques in the Methods section.*
☒	☐	A description of all covariates tested
☐	☒	A description of any assumptions or corrections, such as tests of normality and adjustment for multiple comparisons
☐	☒	A full description of the statistical parameters including central tendency (e.g. means) or other basic estimates (e.g. regression coefficient) AND variation (e.g. standard deviation) or associated estimates of uncertainty (e.g. confidence intervals)
☐	☒	For null hypothesis testing, the test statistic (e.g. F, t, r) with confidence intervals, effect sizes, degrees of freedom and P value noted<br>*Give P values as exact values whenever suitable.*
☒	☐	For Bayesian analysis, information on the choice of priors and Markov chain Monte Carlo settings
☒	☐	For hierarchical and complex designs, identification of the appropriate level for tests and full reporting of outcomes
☐	☒	Estimates of effect sizes (e.g. Cohen's d, Pearson's r), indicating how they were calculated

Our web collection on statistics for biologists contains articles on many of the points above.

Software and code

Policy information about availability of computer code

Data collection	NIS Elements;
Data analysis	ImageJ (FIJI) version 2.0.0-rc-69/1.52p; MATLAB version R2019b; Python 3.7.13; custom scripts for image processing and numerical calculations are described in the methods section and supplementary information and are available upon reasonable request.

For manuscripts utilizing custom algorithms or software that are central to the research but not yet described in published literature, software must be made available to editors and reviewers. We strongly encourage code deposition in a community repository (e.g. GitHub). See the Nature Portfolio guidelines for submitting code & software for further information.

Data

Policy information about availability of data

All manuscripts must include a data availability statement. This statement should provide the following information, where applicable:

- Accession codes, unique identifiers, or web links for publicly available datasets
- A description of any restrictions on data availability
- For clinical datasets or third party data, please ensure that the statement adheres to our policy

Source data are provided with this paper. All other data used in this study are available upon reasonable request from the corresponding author.

Research involving human participants, their data, or biological material

Policy information about studies with human participants or human data. See also policy information about sex, gender (identity/presentation), and sexual orientation and race, ethnicity and racism.

Reporting on sex and gender	N/A
Reporting on race, ethnicity, or other socially relevant groupings	N/A
Population characteristics	N/A
Recruitment	N/A
Ethics oversight	N/A

Note that full information on the approval of the study protocol must also be provided in the manuscript.

Field-specific reporting

Please select the one below that is the best fit for your research. If you are not sure, read the appropriate sections before making your selection.

[x] Life sciences   [ ] Behavioural & social sciences   [ ] Ecological, evolutionary & environmental sciences

For a reference copy of the document with all sections, see nature.com/documents/nr-reporting-summary-flat.pdf

Life sciences study design

All studies must disclose on these points even when the disclosure is negative.

Sample size	All sample sizes detailed in the paper are described as number of biological replicates (i.e. number of distinct extract preparations from different frogs) and number of independent measurements for each biological replicate.
Data exclusions	No relevant data were excluded.
Replication	As specifically stated in the paper for each experiment, all measurements were taken across multiple different biological replicates. All attempts at reproducing results across different biological replicates were successful.
Randomization	N/A
Blinding	N/A

Reporting for specific materials, systems and methods

We require information from authors about some types of materials, experimental systems and methods used in many studies. Here, indicate whether each material, system or method listed is relevant to your study. If you are not sure if a list item applies to your research, read the appropriate section before selecting a response.

Materials & experimental systems

n/a	Involved in the study	
[ ]	[x]	Antibodies
[ ]	[x]	Eukaryotic cell lines
[x]	[ ]	Palaeontology and archaeology
[ ]	[x]	Animals and other organisms
[x]	[ ]	Clinical data
[x]	[ ]	Dual use research of concern
[x]	[ ]	Plants

Methods

n/a	Involved in the study	
[x]	[ ]	ChIP-seq
[x]	[ ]	Flow cytometry
[x]	[ ]	MRI-based neuroimaging

Antibodies

Antibodies used	augmin: His-tagged Xenopus laevis HAUS1 used to produce rabbit polyclonal anti-HAUS1 serum (Genscript);<br>tpx2: GST-tagged Xenopus laevix TPX2 alpha 3-7 used to produce rabbit polyclonal anti-TPX2 serum (Genscript);<br>NuMA: Invitrogen, GT3611, Mouse, monoclonal;<br>Eg5: His-tagged Eg5 fragment used to produce rabbit polyclonal anti-Eg5 serum (Genscript);

	dsDNA: Abcam, ab27156, Mouse, monoclonal;<br>Random IgG: Sigma, I5006, Rabbit, polyclonal;<br><br>All working dilutions are detailed in the methods section.
Validation	augmin: validated by immunodepletion from Xenopus egg extract and by western blot<br>tpx2: validated by immunodepletion from Xenopus egg extract and by western blot<br>NuMA: validated by successful immunofluorescence staining of bulk Xenopus egg extract spindles<br>Eg5: validated by successful immunofluorescence staining of bulk Xenopus egg extract spindles<br>dsDNA validated by successful attachment of purified chromosomes to glass surface<br>Random IgG: validated by successful positive controls of immunodepletion experiments and negative controls of immunofluorescence experiments

Eukaryotic cell lines

Policy information about cell lines and Sex and Gender in Research

Cell line source(s)	HeLa CENPA-GFP
Authentication	Cell lines were not authenticated.
Mycoplasma contamination	Cell lines were not tested for mycoplasma contamination.
Commonly misidentified lines<br>(See ICLAC register)	N/A

Animals and other research organisms

Policy information about studies involving animals; ARRIVE guidelines recommended for reporting animal research, and Sex and Gender in Research

Laboratory animals	Mature (2-7 years old), female, Xenopus laevis frogs were used.
Wild animals	No wild animals were used in this study.
Reporting on sex	N/A
Field-collected samples	No field-collected samples were used in this study.
Ethics oversight	Animal care was done in accordance with recommendations in the Guide for the Care and Use of Laboratory Animals of the NIH and the approved Institutional Animal Care and Use Committee (IACUC) protocol 1941-16 of Princeton University.

Note that full information on the approval of the study protocol must also be provided in the manuscript.
