# Supplementary material for: Acentrosomal spindles assemble from branching microtubule nucleation near chromosomes in Xenopus laevis egg extract
Source: Nat Commun. 2023 Jun 21;14:3696. doi: 10.1038/s41467-023-39041-z (PMC10284841; doi:10.1038/s41467-023-39041-z)
Supplement: Supplementary file 4 — Description of Additional Supplementary files [file 41467_2023_39041_MOESM4_ESM.docx]

**SUPPLEMENTARY MOVIE LEGENDS**

Supplementary Movie 1. Branching microtubule nucleation at kinetochores with vanadate. The field is 30 µm x 30 µm.

Supplementary Movie 2. Branched microtubule network formation in control (left) and augmin-depleted (right) extracts. The fields are 140 µm x 166 µm.

Supplementary Movie 3. Branched microtubule network formation in control (left) and TPX2-depleted (right) extracts. The fields are 140 µm x 166 µm.

Supplementary Movie 4. Branching microtubule nucleation in a uniform field of SAFs. The field is 34 µm x 22 µm.

Supplementary Movie 5. Branching microtubule nucleation at kinetochores with motor activity. The field is 30 µm x 30 µm.

Supplementary Movie 6. Bipolar spindle assembly around chromosomes. The field is 40 µm x 40 µm.
